# The Influence of Impeller Geometries on Hemolysis in Bearingless Centrifugal Pumps

**DOI:** 10.1109/OJEMB.2020.3037507

**Published:** 2020-11-16

**Authors:** Pascal Puentener, Marcel Schuck, Johann W. Kolar

**Affiliations:** Power Electronic Systems Laboratory 8092 Zurich Switzerland; Power Electronic Systems LaboratoryETH Zurich 419061 Physikstrasse 3 8092 Zurich Switzerland

**Keywords:** Centrifugal pumps, extracorporeal life support, hemolysis, in vitro, magnetic levitation

## Abstract

*Goal:* The importance of the main impeller design parameters in bearingless centrifugal pumps with respect to hemolysis for cardiopulmonary bypass (CPB) and extracorporeal membrane oxygenation (ECMO) applications are studied in this work. *Methods:* Impeller prototypes were designed based on theoretical principles. They were manufactured and their hydraulic and hemolytic performance were analyzed experimentally. The cell compatibility is benchmarked against commercially available centrifugal blood pumps BPX-80 (Medtronic) and FloPump 32 (International Biophysics Corporation). *Results:* The developed prototypes outperform the BPX-80 and FloPump 32 with regard to hemocompatibility by more than a factor of 4.5. The implemented pump features reduced overall and priming volumes. A significant improvement of the cell compatibility is achieved by increasing the radial gap between the impeller and the pump head. The blade should be sufficiently high and a blade outlet angle of 90° provides favorable performance. No correlation between the hydraulic and hemolytic performance is observed. *Conclusions:* This work identified the most important geometrical parameters of the impeller for blood pumps with respect to cell compatibility. This provides valuable design guidelines for improving existing pumps.

## I. Introduction

Extracorporeal life support (ECLS) is used during surgery and to support failing organ systems of the human body. During cardiac surgery, the cardiopulmonary bypass (CPB) technique is applied to temporarily take over the function of the heart and lungs. In case of acute respiratory diseases, a modified form of CPB, referred to as extracorporeal membrane oxygenation (ECMO) is used. Compared to CPB, where 100% of the blood flow is directed through the pump, the heart function is only supported and approximately 20% of the volume flow still passes through the heart and lung to prevent stagnation and clotting. Due to the lower stress on these organs, they are able to regenerate more quickly [1].

If both, the heart and lung function, must be supported, blood is drawn venously, filtered, oxygenated, tempered outside the body, and injected arterially (VA-ECMO). If only the lung has to be supported, blood is drawn venously and returned venously (VV-ECMO). During VV-ECMO, the heart continues its normal function. Thus, VA-ECMO is typically used in case of cardiac failure, while VV-ECMO is used in case of respiratory failure [1].

To provide a continuous blood flow through the extracorporeal circuit, specialized blood pumps that are designed to be hemocompatible are required. One of the complications arising from the use of such mechanical circulation is the rupture and destruction of red blood cells (hemolysis) caused by the severe stresses acting on the blood.

The objective of this work is to analyze the importance of different geometrical parameters of the impeller in bearingless centrifugal pumps with respect to hydraulic efficiency and hemolysis by means of experiments. The results are intended to provide design guidelines for improving the cell compatibility of blood pumps and ultimately result in higher survival rates in future ECLS applications. The findings of this study are particularly relevant for bearingless centrifugal pumps. Their applicability to other pump and motor concepts with significantly different geometries, dimensions, and operating conditions needs to be carefully assessed. In vitro testing of newly developed pumps remains necessary.

### A. State of the Art

Until 2009, roller pumps were the predominant type of pump used in ECLS [1]. Because of their difficult handling and the risk of tube rupture, roller pumps were replaced by centrifugal pumps, which allow for longer continuous pump operation and provide the ability to occlude the circuit tubing.

The Bio-Medicus vortex pump (presently Medtronic BPX-80) was the first centrifugal pump to be tested as an ECLS pump in 1975 and brought to the market in the same year [1]. This pump relies on a pivot bearing that is likely its main source for cell destruction due to local heat generation [2]. In the following years, several other centrifugal pumps with a pivot bearing were brought to market. Many of them, such as the Rotaflow (Maquet), Revolution (Livanova), Affinity (Medtronic), and Deltastream (Medos), are still available today [1].

In 2004, the CentriMag (Abbott) was the first pump featuring a completely magnetically suspended impeller that was clinically tested [3]. The impeller contains a permanent magnet and is levitated without mechanical contact in the center of the pump housing. The position of the impeller is stabilized by means of actively controlled electromagnets.

In the paper at hand, the same bearingless motor type is used. It combines the bearing and drive unit in a single magnetic circuit, thus resulting in a highly compact system. The rotor features a disk shape, which allows for passive stabilization in three degrees of freedom, namely with respect to axial displacements and tilting around the axis of magnetization and perpendicular to it. Only the two radial degrees of freedom have to be controlled actively to attain stable levitation [4].

**Fig. 1.**
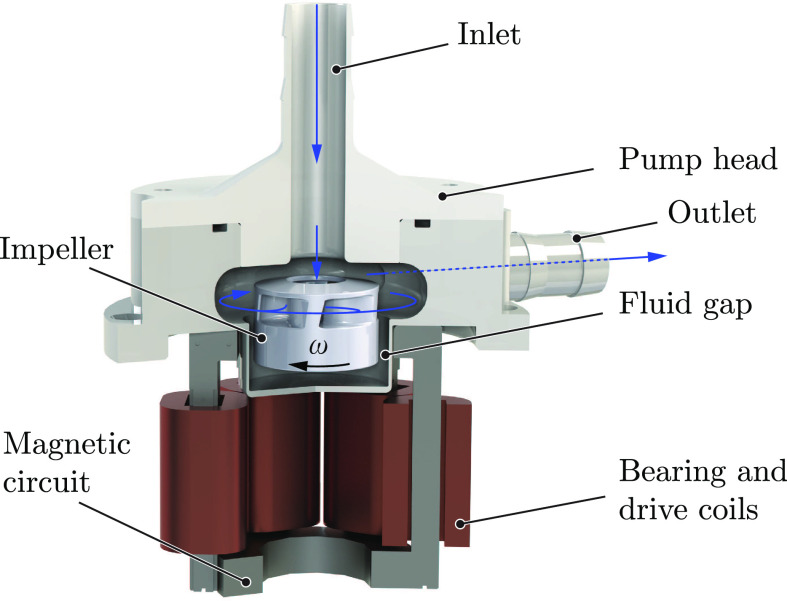
Cross-sectional view of a bearingless pump including the pump head, impeller, and main components of the magnetic circuit.

Fig. 1 shows a cross-sectional view of such a bearingless pump. Due to the magnetic suspension, the impeller is entirely surrounded by the fluid. No mechanical contact between the impeller and the housing occurs, resulting in large fluid gaps for the blood cells. Thus, shear forces caused by narrow gaps and high relative speeds can be decreased and stagnant zones are reduced to avoid thrombus formation.

### B. Blood Damage in Mechanical Circulatory Support

Due to its complexity, the process of cell damage is still not entirely understood. However, a broad consensus in the literature exists that cell damage is related to shear forces [5]. Several studies have explored the relation between the shear stress and exposure time on the destruction of red blood cells (RBCs) [5]–[8]. Several experiments show that a threshold value of around ${250-425}$ Pa exists at which hemolysis starts to occur [8]–[10]. However, sublethal damages that lead to a reduced deformability may already occur at shear stresses as low as 30 Pa [11]. These experiments were conducted using specifically designed devices, such as Couette viscometers, that exhibit rather simple flow conditions. While these studies provide valuable indications for pump designers, the considered simple flow conditions cannot capture all hemolysis-generating effects in a rotary pump. Therefore, it is necessary to investigate these conditions and the pump performance separately.

Several studies exist that benchmark commercially available products and prototypes against each other [12]–[18]. The considered pumps usually differ significantly in their design or even their underlying concepts. To date, only few studies aimed at identifying design rules by systematically modifying design parameters of a single pump type exist. Noteworthy approaches were carried out for pressure differences typical for ventricular assist device (VAD) applications (100 mmHg) [19]–[24]. Similarly, experiments at higher pressures, as typical for extracorporeal applications, can be found in [21], [22], [25]–[27]. The considered pumps all feature pivot bearings that result in some mechanical contact between the impeller and the housing.

In the study at hand, in vitro hemolysis experiments on selective impeller variations for a fully magnetically levitated pump for ECLS applications are conducted. To the authors' knowledge, this is the first in vitro study that considers impeller geometry variations for this type of pump.

## II. Materials And Methods

### A. Pump Design

#### 1) Operating Conditions

During VA-ECMO, the amount of flow that is taken over from the heart by the pump depends on the severity of the disease. The highest flow rates have to be provided for severe diseases and high oxygenation, as well as during CPB, where the entire flow of the human heart (on average $Q=5$ l/min) has to be covered.

The required pressure depends on the circuit and the applied ECMO technique. While the average difference between the systolic and diastolic pressure is around $\Delta p=100$ mmHg for adults, the pressure required for ECMO is in the range of $\Delta p = \text{400}\,\text{mmHg}$ to overcome the resistances of the oxygenator and heat exchanger. For VV-ECMO, the pressure difference is lower, as the blood is returned to the venous circulation.

For this paper, the pump was designed and tested for $Q_{\mathrm{nom}}=5$ l/min and $\Delta p_{\mathrm{nom}}=350$ mmHg as recommended by the ASTM standard [28]. As the flow rate and required pressure depend on the size of the human body and the application, the pump must cover a broad range of pressures and flows. The presented prototypes are designed for a maximum flow rate of 9 l/min at nominal pressure and a maximum pressure of 700 mmHg at nominal flow to provide sufficient margins.

#### 2) Euler Equation

**Fig. 2.**
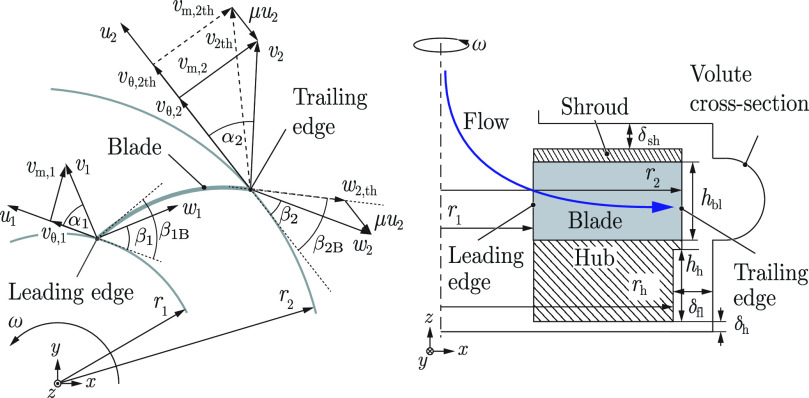
Cross-sectional top (left) and side (right) view of an impeller with velocity triangles at the blade inlet (subscript 1) and outlet (subscript 2), adapted from [29].

In centrifugal pumps, the pressure is generated by means of centrifugal forces. The fluid enters the impeller blade at the top opening around the axis of rotations in axial direction and exits the blade passage at the circumference of the impeller in radial direction towards the volute. Fig. 2 shows a cross-sectional view of such an impeller passage. In a first step, it is sufficient to only consider the inlet and outlet of the blade passage to analyze the behavior of the pump. According to the Euler's turbine equation, the specific work of a centrifugal pump can be calculated as

\begin{equation*}
Y_\text{th} = \frac{\Delta p_\text{th}}{\rho } = u_2 v_{\theta, 2\text{th}} - u_1 v_{\theta, 1\text{th}}. \tag{1}
\end{equation*}

Using geometric dependencies, assuming zero inlet swirl, and considering some leakage flow, the specific work can be expressed as

\begin{equation*}
Y_\text{th}=(\omega r_2)^2-\frac{\omega Q_\text{bl}}{2\pi h_\text{bl} \tan (\beta _\text{2B})}, \tag{2}
\end{equation*}

where $Q_{\mathrm{bl}}=Q_{\mathrm{net}}/\eta _{\mathrm{vol}}$, $\eta _{\mathrm{vol}}$, and $h_{\mathrm{bl}}$ denote the flow through the blade passage, the volumetric efficiency, and the blade height, respectively. Due to the pressure difference between the pressure side and the suction side of the blade, the flow at the trailing edge will deviate from the blade angle $\beta _{\mathrm{2B}}$ by $v_{\theta,2\text{th}} - v_{\theta,2} = \mu u_2$, where the slip factor $\mu$ can be estimated as

\begin{equation*}
\mu = 1 - f_1 \left(1 - \sqrt{\sin (\beta _{\mathrm{2B}})} \right) k_{\mathrm{W}}, \tag{3}
\end{equation*}

with $k_{\mathrm{W}} = 1$ and $f_1 = 0.98$ for radial pump designs [30]. This results in an estimated specific work of

\begin{equation*}
Y = (1-\mu )(\omega r_2)^2 - \frac{\omega Q_{\mathrm{bl}}}{2 \pi h_{\mathrm{bl}} \tan (\beta _{\mathrm{2B}})}. \tag{4}
\end{equation*}

#### 3) Cordier Diagram and Impeller Diameter

The optimal impeller diameter with respect to hydraulic efficiency for a given operating point (flow rate, pressure, and rotational speed) can be found based on an empirical study of industrially used turbomachinery [30]. In the work at hand, the Cordier diagram [31] was used to determine a suitable impeller diameter for which a radial impeller design achieves the highest efficiency. Based on the nominal pressure $\Delta p_{\mathrm{nom}}$ and fluid density ${\rho = \text{1060}\,\text{kg/m}^3}$, the specific work

\begin{equation*}
Y = \frac{\Delta p_{\mathrm{nom}}}{\rho } = 44.02\,\frac{{\rm m}^2}{{\rm s}^2}, \tag{5}
\end{equation*}

can be calculated. The diameter number $\delta _{\mathrm{M}}$ is then related to the impeller diameter $d_2 = 2 r_2$ as

\begin{equation*}
\delta _{\mathrm{M}} = d_2 \frac{\sqrt{\pi }}{2} \frac{(2Y)^{1/4}}{Q^{1/2}}. \tag{6}
\end{equation*}

According to the Cordier diagram, radial impellers are best suited for diameter numbers $\delta _{\mathrm{M}} > 3.5$. Any impeller diameter $d_2 > \text{11}\,\text{mm}$ fulfills this criterion. To reduce the effect of manufacturing tolerances, a larger diameter of $d_2 = \text{22}\,\text{mm}$ was chosen, corresponding to a diameter number of $\delta _{\mathrm{M}} = 6.54$. This results in a specific speed of

\begin{equation*}
\sigma _{\mathrm{M}} = \frac{\omega }{\sqrt{\pi }}\frac{Q^{1/2}}{(2Y)^{3/4}} \approx 0.137, \tag{7}
\end{equation*}

coinciding with a rotational speed of $n = \text{7310}\,\text{rpm}$. A larger impeller also simplifies the control of the magnetic bearing.

#### 4) Outlet Angle and Blade Height

**Fig. 3.**
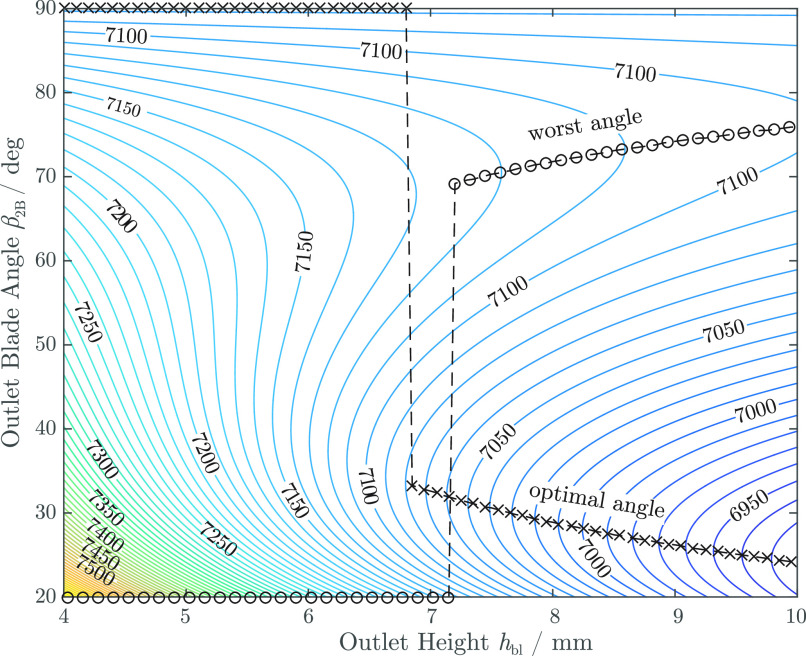
Required theoretical rotational speed $n$ (in $\text{rpm}$) to achieve the nominal operating point for different blade outlet angles $\beta _{\mathrm{2B}}$ and heights $h_{\mathrm{bl}}$. The optimal and worst combination of parameters are indicated by crosses and circles, respectively.

The outlet angle $\beta _{\mathrm{2B}}$ and blade height $h_{\mathrm{bl}}$ of the impeller cannot be chosen independently. With increasing values of $h_{\mathrm{bl}}$, the meridional velocity $v_{\mathrm{m,2}}$ decreases while $v_{\mathrm{\theta,2}}$ increases. Typically, a pump design starts with an outlet angle close to $\beta _2 = 22.5^\circ$ [30], [32]. For the design presented in this work, an outlet angle of $\beta _2 = 90^\circ$ was chosen. A higher outlet angle generally results in a flatter characteristic curve but leads to an increased slip coefficient. The theoretical speed that is required to achieve the nominal operating point can be calculated as a function of $h_{\mathrm{bl}}$ and $\beta _{\mathrm{2B}}$ based on equation (4) (see Fig. 3).

The lowest rotational speeds are obtained for low outlet angles and large outlet heights. However, the required speed is highly sensitive to the outlet height. For $h_{\mathrm{bl}} < \text{6.8}\,\text{mm}$, a blade angle of $\beta _{\mathrm{2B}} = 90^\circ$ becomes optimal with respect to a decrease of the rotational speed and the benefits of a low outlet angle are small for $h_{\mathrm{bl}} > \text{6.8}\,\text{mm}$. Furthermore, an angle of $90^\circ$ results in a pump head that is almost independent of the blade height. This improves the comparability of different height variations, as all designs will theoretically require the same rotational speed and, therefore, generate similar shear forces in the radial gap. Thus, a more isolated parameter variation is possible. Blade angles above $90^\circ$ are not considered as they lead to $\mathrm{d}(\Delta p)/\mathrm{d}Q > 0$, which might cause unstable operation.

For the aforementioned calculations, a volumetric efficiency of $\eta _{\mathrm{vol}} = 0.4$ was assumed. While this might seem rather low, an efficiency of no more than $\eta = 0.6$ can be expected for such small pumps and flow rates [30]. Additionally, the gaps surrounding the impeller have to be sufficiently large to ensure that the impeller does not touch the housing. This leads to increased leakage flows and, consequently, lowered volumetric efficiency.

#### 5) Eye Radius

The circular opening at the inlet side of the impeller, referred to as the eye, is designed such that a minimal relative velocity between the fluid and the leading edge of the blade is achieved. Considering the velocity triangle at the inlet, the relative velocity $w_1$ can be expressed as

\begin{equation*}
w_1 = \sqrt{v_{\mathrm{m,1}}^2 + \left(u_1 - \frac{v_{\mathrm{m,1}}}{\tan (\alpha _1)} \right)^2}, \tag{8}
\end{equation*}

where

\begin{equation*}
v_{\mathrm{m,1}} = \frac{Q_{\mathrm{bl}}}{\pi r_1^2} \quad \text{and} \quad u_1 = r_1 \omega. \tag{9}
\end{equation*}

The required eye radius $r_1$ is found by taking the derivative of equation (8) with respect to $r_1$ and obtaining the roots of the resulting polynomial as

\begin{equation*}
{r_\mathbf {1} = \sqrt[3]{ \frac{Q_{\mathrm{bl}}}{4\omega \pi } \left(\sqrt{2 (17 + \cos (2\alpha _1))} \frac{1}{\sin (\alpha _1)} -\frac{2}{\tan (\alpha _1)} \right) }}. \tag{10}
\end{equation*}

For a volumetric efficiency of $\eta _{\mathrm{vol}} = 0.4$, and inlet radius of $r_1 = \text{5}\,\text{mm}$ results.

#### 6) Volute Design

A conventional semi-volute design is chosen to reduce the radial thrust over the entire range of the flow-rate. This also prevents regions of high shear stress at the volute tongue. The radial distance from the impeller to the outer wall of the volute is chosen larger than in classical pump designs to reduce the circumferential velocity of the flow near the volute housing. These are typical design choices for slurry pumps [32] that are assumed to reduce hemolysis.

The inlet and outlet are chosen to have an inner diameter of $3/8^{\prime \prime }$ according to the common tube diameter for $Q=5\,\text{l/min}$ during ECLS [1]. The meridional cross-sectional area of the volute is chosen equal to the pressure socket to avoid acceleration of the fluid towards the outlet. The priming volume of the resulting design is $\text{15.5}\,\text{ml}$.

### B. Tested Impeller Variations

To identify the most important geometric parameters with respect to cell damage, the major design parameters, namely the blade outlet angle $\beta _{\mathrm{2B}}$, blade height $h_{\mathrm{bl}}$, blade radius $r_2$, radial gap $\delta _{\mathrm{fl}}$, and hub depth $h_{\mathrm{h}}$ (see Fig. 2) were adjusted and tested. In addition, an impeller with a semi-open shroud and long blades ($r_2 > r_{\mathrm{h}}$), reaching halfway into the cross-section of the volute, was tested.

Table I lists all tested impeller variations including the geometric regions that were analyzed and the corresponding altered parameters. The data stated in percent relates to the base design B which is designed according to Section II-A with a blade height $h_{\mathrm{bl}} = \text{6}\,\text{mm}$ and hub height $h_{\mathrm{h}} = \text{8}\,\text{mm}$. The axial stiffness of the passive magnetic bearing is denoted by $k_{\mathrm{z}}$. Some impeller variations result in an undesired influence on other geometric parameters, e.g. a larger blade height $h_{\mathrm{bl}}$ additionally reduces the top gap $\delta _{\mathrm{sh}}$ between the impeller lid and the housing.

**TABLE I:** Tested Geometry Variations of the Impeller

	$h_{\mathrm{bl}}$ ($\%$)	$h_{\mathrm{h}}$ ($\%$)	$r_2$ ($\%$)	$\delta _{\mathrm{fl}}$ ($\text{mm}$)	$\beta _{\mathrm{2B}}$ ($^\circ$)	Addressed effect	Coupled parameters
B	100	100	100	0.65	90	base design	-
BH+	**116**	100	100	0.65	90	blade height	$\delta _{\mathrm{sh}}$
BH−	**83**	100	100	0.65	90	blade height	$\delta _{\mathrm{sh}}$
A60	100	100	100	0.65	**60**	blade angle	-
RG+	100	100	**95**	**1.15**	90	diameter	$\omega$, $k_{\mathrm{z}}$
D-	100	100	100	**1.15**	90	radial gap	$k_{\mathrm{z}}$
SO	100	100	**136**	0.65	90	long blades	shroud
HH	100	**80**	100	0.65	90	hub area	$k_{\mathrm{z}}$, $\delta _{\mathrm{h}}$

Between designs B, BH−, and BH+, the blade height $h_{\mathrm{bl}}$ was varied with the intention of analyzing the influence of the relative fluid velocity $w$ within the blade passage, where the highest pressures occur. Higher values of the blade height lead to lower relative velocities within the blade passage. A variation of the blade outlet featuring a lower angle $\beta _{\mathrm{2B}}$ of $60^\circ$ instead of $90^\circ$ is covered by the design A60. The influence of the radial fluid gap is addresses by the designs D- and RG+. While only the radial gap is increased for RG+, the effect of a smaller overall impeller diameter is covered by D-. According to equation (4), the two designs require different rotational speeds $\omega$, while both feature identical dimensions of the radial gap. The semi-open design SO features an increased blade radius $r_2$, reaching into the volute. Due to the increased blade radius, the rotational speed can be decreased and, thus, the shear stress within the radial gap can be reduced significantly. To prevent narrow gaps between the shroud and hub towards the volute walls, this impeller is designed with a semi-open blade. The shroud and hub diameters are equal to the base design and only the blades are enlarged towards the volute center. Finally, HH- exhibits a reduced hub height $h_{\mathrm{h}}$ to decrease the amount of fluid volume exposed to the high shear stresses in the radial gap. Otherwise, this impeller equals the base design.

All impellers and housings were milled from polycarbonate with minimum possible surface roughness ($Ra \approx 0.8$). To further improve the quality of the parts, they were vapor polished using Dichloromethane gas, thereby achieving an average surface roughness of $Ra \approx 0.05$.

### C. Hydraulic Performance Testing

To test the hydraulic performance of the impellers, an automated circuit was used. The pump was connected to a reservoir with two electromechanically actuated valves in parallel. During operation, the fluid was tempered to $40\,^\circ {\rm C}$ to ensure a constant temperature for all operating points. The inlet and outlet pressure were measured approximately $\text{30}\,\text{cm}$ up- and downstream of the pump. The flow rate was measured downstream using an ultrasonic flow sensor.

The hydraulic efficiency of the pump was calculated according to

\begin{equation*}
\eta = \frac{P_{\mathrm{hyd}}}{P_{\mathrm{in}} - P_{\mathrm{iron}} - P_{\mathrm{ohm}} - P_{\mathrm{ctrl}}}, \tag{11}
\end{equation*}

where $P_{\mathrm{hyd}}$, $P_{\mathrm{in}}$, $P_{\mathrm{iron}}$, $P_{\mathrm{ohm}}$, and $P_{\mathrm{ctrl}}$ represent the hydraulic power measured within the tube, the electrical input power, stator iron losses, conduction losses, and losses related to the control circuit of the pump, respectively. A separation of the pump efficiency into its mechanical, hydraulic, and volumetric components is not possible as the flow conditions within the pump cannot be measured.

### D. In Vitro Hemolysis Testing

#### 1) Blood Collection and Preparation

For the presented in vitro study, bovine blood was used as its hemolytic characteristics are similar to those of human blood [33]. Fresh blood was collected from a local slaughterhouse, mixed immediately with $\text{8000}\,\text{IU/l}$ anticoagulant and transported in a sealed food-grade canister. The blood was then diluted to a hematocrit of $Hct = 30\%$ using phosphate-buffered saline and filtered using a blood filter. All equipment was rinsed with phosphate-buffered saline prior to its contact with blood. The experiments were started within one hour after collecting the blood.

#### 2) Circuit

**Fig. 4.**
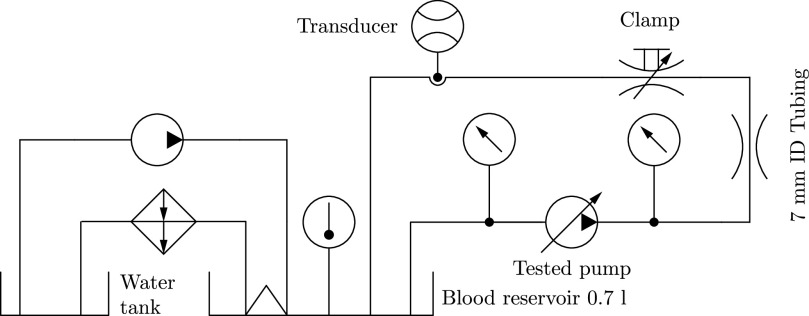
Pump circuit used for hemolysis testing including all necessary elements. Refer to the text for details.

Each pump circuit, as shown in Fig. 4, was operated with a total circuit volume of $V=700\,\text{ml}$ using a PVC blood reservoir. A heating coil was placed in the reservoir to temper the blood to $30\,^\circ \text{C}$. The flow rate was measured using a clamp-on transducer at the high pressure side near the reservoir inlet. The pressure was measured $\text{20}\,\text{cm}$ up- and downstream of the pump using digital manometers. The desired pressure drop was achieved by a $\text{2.9}\,\text{m}$ long tube with an inner diameter of $\text{7}\,\text{mm}$ at the high-pressure side. An additional clamp, located at the high-pressure side, was used to fine adjust the operating conditions to be within the ASTM tolerances ($Q= 5\pm \text{0.25}\,\text{l/min}$, $\Delta p = 350\pm \text{10}\,\text{mmHg}$) [28]. The inlet tubing was kept short ($\text{40}\,\text{cm}$ of $\text{10}\,\text{mm}$ ID tubing) to attain sufficient inlet pressure to prevent cavitation within the pump. The mean pressure measured at the inlet was $-17\,\text{mmHg}$. A control volume at rest was tested in parallel to monitor the blood conditions and to detect potential irregularities.

#### 3) Measurement

Blood samples of $\text{5}\,\text{ml}$ were removed from the reservoir at an interval of $\text{1}\,\text{h}$. The samples were centrifuged at $\text{5000}\,\text{rpm}$ for $\text{10}\,\text{min}$. To ensure a clear separation of the plasma, $170\,\mu \text{l}$ of plasma were centrifuged a second time. The resulting plasma was diluted with $\text{2}\,\text{ml}$ of Drabkin's reagent, mixed using a tube shaker, and allowed to react for $\text{15}\,\text{min}$. The amount of plasma free hemoglobin ($pfHb$) was obtained by measuring the absorption of the dilution at a wavelength of $\text{540}\,\text{nm}$ [34]. To relate the absorption to the amount of $pfHb$, a calibration curve was obtained using dried bovine erythrocytes. To evaluate the different experiments, the normalized index of hemolysis ($NIH$) is calculated according to

\begin{equation*}
NIH = \frac{\Delta pfHb}{\Delta t} \cdot \frac{V}{Q} \cdot \frac{100 - Hct}{100}, \tag{12}
\end{equation*}

where the hematocrit $Hct$ needs to be inserted in percent. The generation of plasma free hemoglobin over time $\Delta pfHb/\Delta t$ is approximated by a linear regression.

To reduce the variation between different experiments caused by the blood sensitivity of different individuals, the industrial bearingless pump DCP-1.4 (Levitronix GmbH) was run simultaneously during all experiments, acting as an independent reference. The different designs can thus be compared to the reference value $NIH_{\mathrm{ref}}$.

## III. Results

### A. Hydraulic Tests

**Fig. 5.**
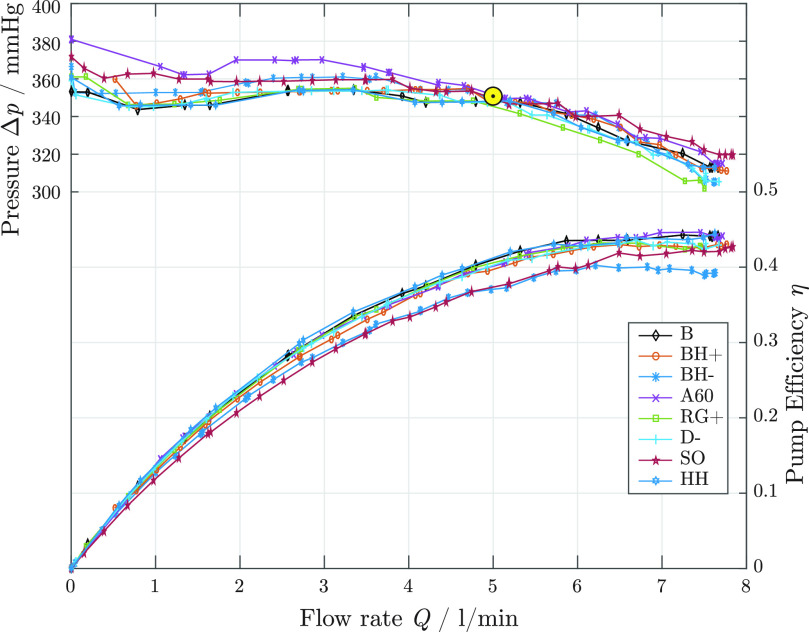
Pump characteristics and efficiencies for all tested impeller geometries. The nominal operating point is marked in yellow.

All impeller designs were tested hydraulically at their nominal operating speeds. The resulting pressure-flow curves, including the pump efficiencies $\eta$ are shown in Fig. 5. The indicated points represent the average value over ten measurements. All impellers exhibit a flat characteristic curve and reach the nominal operating point. Below $\text{2.5}\,\text{l/min}$ most impellers exhibit a small static instability ($\mathrm{d}(\Delta p)/\mathrm{d}Q>0$) due to recirculation losses. The efficiency depends on the impeller geometry and is summarized in Table II. The designs featuring long blades with a semi-open shroud (SO) and low blade heights (BH−) show the lowest efficiency of $\eta = 0.37$. The other designs perform similarly well, while the highest efficiency of $\eta = 0.41$ is reached by HH- (reduced hub height).

**TABLE II:** Results of the Hemolytic in Vitro Tests

Design	Mean $NIH$ ($mg/dl$)	Std $NIH$ ($mg/dl$)	Mean $\frac{NIH}{NIH_{\mathrm{ref}}}$	Std $\frac{NIH}{NIH_{\mathrm{ref}}}$	$n$ ($rpm$)	$\eta$ ($\%$)
B	0.0096	0.0043	0.625	0.161	7880	40.1
BH+	0.0081	0.0151	0.605	0.180	7780	39.6
BH−	0.0180	0.0215	0.948	0.305	8060	37.1
A60	0.0106	0.0042	0.978	0.439	8050	40.1
D-	0.0059	0.0025	0.500	0.207	8330	40.5
RG+	0.0131	0.0142	0.458	0.140	7820	40.3
SO	0.0239	0.0329	0.933	0.342	5640	37.1
HH-	0.0130	0.0058	0.850	0.289	7920	41.3

Except for the design SO, all prototypes reach the operating conditions listed in Section II-A1. The impeller SO requires too high of a drive current to reach a pressure of $\text{700}\,\text{mmHg}$ and shows instabilities in the axial direction at high volumetric flows, causing the blade tips to touch the housing at the volute.

### B. Benchmark Test

To relate the cell compatibility of the developed prototype to state-of-the-art pumps, the standard impeller design was benchmarked against the commercially available BioPump BPX-80 (Medtronic) and the FloPump 32 (International Biophysics Corporation), which is identical to the Maquet Rotaflow, using the test setup described in Section II-D. For the commercial pumps, new pump heads were used for each experiment, whereas the developed prototypes were reused after cleaning and sterilization. The obtained results are listed in Table III.

**TABLE III:** Benchmarking Results of the Base Design B Against the BPX-80 and FloPump 32

Pump	NIH ($mg/dl$)	Std ($mg/dl$)	$p$	$N$	Volume ($ml$)	$n$ ($rpm$)
B	0.0096	0.0043	-	16	15.5	7900
BPX-80	0.0502	0.0422	0.001	14	80	3140
FloPump 32	0.0261	0.0141	0.058	5	32	3830

The base design B shows a statistically significant improvement of the hemocompatibility compared to the BPX-80 and FloPump 32 ($p = 0.0014$ and $p=0.0575$, respectively, for a two-sample $t$-test, assuming unequal variances of the populations) with the mean $NIH$ being approximately 4.5 times lower. At the same time, the priming volume is more than 5 times smaller.

### C. Hemolysis Tests

All impeller variations were tested for their cell compatibility according to Section II-D. For each experiment, four prototypes and the reference pump were tested simultaneously.

**Fig. 6.**
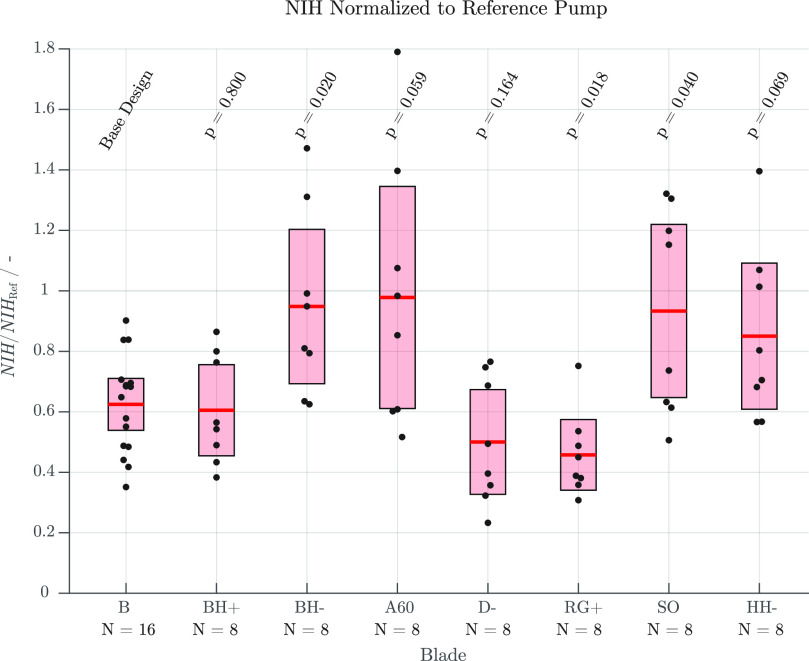
$NIH$ values normalized to the reference pump of all tested pumps including the observed sample mean (red line) and its 95% $t$-interval (red bar). The $p$-values from a two-sample $t$-test with unequal variance compared to the base design B are provided near the top of the plot.

Each variation was tested 8 times. The base design was tested 16 times in total. Fig. 6 summarizes the results of all conducted experiments. The resulting $NIH$ values were normalized to the reference value $NIH_{\mathrm{ref}}$ and evaluated assuming a Student's $t$-distribution with unequal variance and are marked by a red line. The $p$-values of two-sample $t$-tests to the base design B are listed above the 95% $t$-intervals, where the latter are shown by boxes. The assumed null-hypothesis is that the two means result from the same $t$-distributions (i.e. the pumps cause equal hemolysis). Table II lists the absolute values of the observed mean $NIH$ values, their normalized version, and the rotational speeds required to achieve the nominal operating point during the in vitro experiments.

## IV. Discussion

In the following, the results of the hemolysis tests are discussed in detail and interpreted.

### A. Blade Height

The impact of the blade height is assessed by comparing the mean values achieved by the designs B, BH−, and BH+ in Fig. 6. While no significant difference in hemocompatibility between the base design B and the design with increased blade height BH+ ($p=0.8$) is observed, the design with decreased blade height BH− ($p=0.02$) exhibits increased hemolysis. The reduced hemocompatibility of the design BH− is likely caused by the lower volumetic efficiency. Due to the lower blade height, the axial gap between the shroud and the top wall of the housing $\delta _{\mathrm{sh}}$ is increased. As a result, the leakage flow is increased, which causes higher losses and an increased volumetric flow $Q_{\mathrm{bl}}$ through the blades as well as higher relative velocities $w$ within the blade passage. Furthermore, a higher number of RBCs has to pass the blade passage multiple times before reaching the outlet, thus experiencing increased damage with each pass. The marginal difference between the designs BH+ and B might be interpreted as a slight improvement due to the higher blade height and, thus, lower relative velocity $w$ in the blade passage. However, the $t$-test shows a $p$-value of $p=0.8$. Thus, the two impeller variations do not differ sufficiently to make a reliable statement. Therefore, it cannot be assumed that increasing the blade height results in a further improvement the hemolytic performance.

### B. Radial Gap

In the designs D- and RG+, the radial gap between the impeller hub and the volute housing was increased with respect to the base design B. Fig. 6 indicates that this larger radial gap leads to a significant improvement of the cell compatibility ($p$-values of 0.164 and 0.018, for the designs D- and RG+, respectively). For the design RG+, the angular velocity is equal to that of the base design B while the shear stress in the radial gap is reduced due to the increased fluid gap $\delta _{\mathrm{fl}}$. The design variation D- exhibits a reduced hemolysis generation despite the necessity to increase the circumferential speed, thus resulting in similar shear stresses in the radial gap as design B. This observation suggests that the reduction in hemolysis is mainly due to the increased washout flow through the radial gap and, therefore, a reduced residence time in regions of high shear stress.

### C. Blade Angle

Compared to the initial design, no improvement of the hydraulic efficiency can be found for a blade angle of $60^\circ$ (design A60), while the cell compatibility is decreased ($p=0.059$). This is likely due to the higher circumferential speed that is required to achieve the operating point and the resulting increased shear stress within the radial gap. As already observed in [27], a blade angle closer to $90^\circ$ seems to be more suitable, which holds for bearingless pumps as well. Nevertheless, angles resulting in a better performance might exist between $60^\circ$ and $90^\circ$.

### D. Long Blades

The semi-open blade of design SO results in a low hydraulic efficiency. This is likely due to the increased slip at the hub and shroud side of the open impeller part. Even though the rotational speed and, thus, the shear stress in the bottom part of this impeller are significantly reduced compared to the base design B, the hemolysis generation significantly increases (${p=0.04}$). This might be caused by the increased shear stresses acting on the fluid near the top, bottom and trailing edges of the open blade part or the additional narrow gaps that are introduced between the impeller and the volute. Furthermore, unstable operating points at high rotational speeds could also negatively affect other operating conditions. Thus, the impeller blades should be shrouded whenever possible.

### E. Hub Height

Design HH- only differs from design B by a reduced magnet and hub height. During hydraulic tests, this impeller performed best. However, it causes more hemolysis ($p=0.069$). As there are no geometric differences to the base design except for the increased volume below the impeller, the increased residence time in this area is likely the reason for additional hemolysis.

### F. Hydraulic Efficiency

Based on the obtained experimental results for the hydraulic efficiency and hemolytic performance, it can be observed that it is not sufficient to design the impeller blades for high hydraulic efficiency as only a weak correlation between these two parameters can be observed. For example, designs D- and RG+ both have an improved cell compatibility compared to B but exhibit similar efficiencies. Between the mean $NIH/NIH_{\mathrm{ref}}$ and the hydraulic efficiency $\eta$, a Pearson's correlation coefficient of only $\rho = -0.5$ can be observed.

### G. Benchmark

For the base design B a cell damage of less than 1/5 of that caused by the BPX-80 pump was observed during in vitro tests ($p=0.01$). Compared to the FloPump 32, the prototype causes less than half the hemolysis ($p=0.058$). Therefore, the developed prototypes show a superior cell compatibility. Furthermore, the higher rotational speed of the impeller leads to significantly reduced overall dimensions. The priming volume of the constructed prototypes is $\text{15.5}\,{\rm ml}$. This is approximately half of the volume required by state-of-the-art pumps (Rotaflow: $\text{32}\,\text{ml}$) and comparable to pediatric versions of commercially available pumps.

## V. Conclusion

A bearingless centrifugal pump was designed and implemented. Several impeller designs were tested hydraulically and with regard to hemolysis. An overall hydraulic efficiency between $\eta = 0.37$ and $\eta = 0.41$ was achieved at the nominal operating point of $Q = \text{5}\,\text{l/min}$ and $\Delta p = \text{350}\,\text{mmHg}$. Best cell compatibility was achieved by an impeller blade with increased radial gap. Further improvements can be achieved with a large blade height.

Impeller blades should not reach into the volute as this reduces the hydraulic efficiency and cell compatibility. No relevant correlation between the hydraulic efficiency and hemolysis was observed.

The developed prototype outperforms the BPX-80 and FloPump 32 pumps in terms of hemolysis generation by a factor of 5.4 and 2.7, respectively and requires a priming volume of only $\text{15.5}\,\text{ml}$. The presented results provide new insights for the design of the latest generation of blood pumps.
